# Attention Affecting Response Inhibition in Overweight Adults with Food Addiction

**DOI:** 10.3390/bios15030180

**Published:** 2025-03-13

**Authors:** Xiaotong Liu, Guangying Pei, Jiayuan Zhao, Mengzhou Xu, Lizhi Cao, Jian Zhang, Tiantian Liu, Jinglong Wu, Shintaro Funahashi, Lei Ding, Li Wang

**Affiliations:** 1 School of Mechatronical Engineering, Beijing Institute of Technology, Beijing 100081, China; 2 School of Medical Technology, Beijing Institute of Technology, Beijing 100081, China; 3 Advanced Research Institute of Multidisciplinary Science, Beijing Institute of Technology, Beijing 100081, China; 4 Key Laboratory of Carcinogenesis and Translational Research (Ministry of Education/Beijing), Department of Anesthesiology, Peking University Cancer Hospital & Institute, Beijing 100142, China

**Keywords:** food addiction, overweight, obesity, attention, response inhibition, EEG microstate

## Abstract

Food addiction is associated with attention bias and response inhibition deficits, while the relationship between these two domains is unclear. Participants with body mass index (BMI) ≥ 25 and exhibiting food addiction behaviors (FA group, n = 20) were compared with healthy controls (HC group, n = 23). We examined attention-inhibition mechanisms using resting EEG microstate analysis, food-cue-evoked event-related potentials (ERPs), and non-food Go/No-Go tasks. Overweight individuals with food addiction behaviors demonstrated attentional deficits, as indicated by abnormalities in microstate D and the P100 component. Importantly, both microstate D and the P100 component significantly predicted No-Go performance, linking neurophysiological markers to behavioral inhibition. This study suggests that attention bias may be an important interaction factor of response inhibition, providing novel mechanistic insights into food addiction.

## 1. Introduction

Food addiction is a risk factor and specific phenotype for overweight and obesity [1,2,3]. The prevalence of food addiction in the overweight or obese population is up to 24.9%, while that in normal-weight individuals is only 11% [4]. As the main treatment for food addiction, the application of cognitive behavioral therapy is limited by slow onset of action and uncertain efficacy. Non-invasive neuromodulation is showing great promise in the treatment of food addiction, owing to its ability to non-invasively modulate cortical activity [5]. Nevertheless, its application is immature due to a lack of knowledge on the neural circuit and electrophysiological mechanisms of food addiction [6].

Response inhibition is a core pathological sign in addicted populations, typically assessed via Go/No-Go tasks. Individuals with excessive eating behaviors exhibit impaired food-specific response inhibition [7,8,9], which is associated with a higher body mass index (BMI) [10]. Notably, general response inhibition—as measured by non-food-specific tasks—does not appear to be significantly impaired [11], particularly in non-clinical populations with food addiction symptoms [12,13]. However, behavioral interventions for overeating and obesity frequently target both food-related attention allocation and response inhibition concurrently [14], suggesting that an attentional bias may be an abnormal feature of food addiction. Indeed, while overweight individuals may not exhibit broad deficits in response inhibition, their enhanced neural processing in response to food cues highlights an attentional bias that underpins abnormal cue reactivity [15,16,17]. In addiction research, the P100 component is often used to examine attention bias to addiction-related cues [18]. The P100 component, involved in early-stage visual attention processing [19], is disrupted in individuals with food addiction [20,21], smoking [22,23], and alcohol dependence [24]. The P100 component is a typical biomarker for verifying early attentional abnormalities in response to food cues in food-addicted individuals.

Resting-state neuroimaging studies in individuals with obesity or food addiction reveal abnormalities, particularly in attentional processes. In individuals with binge eating disorder (BED) comorbid with obesity, increased beta band activity in the fronto-central regions is observed, which correlates with attentional abnormality [25]. fMRI studies further reveal abnormal activation in the dorsolateral prefrontal cortex (dlPFC), a key hub in the attention network [26,27]. Resting-state EEG microstate analysis captures the dynamic spatiotemporal features of brain networks, offering a more comprehensive understanding of brain activity during these processes [28]. Previous studies have presented four microstate categories (i.e., microstate A, microstate B, microstate C, microstate D) that can explain most of the abnormalities in cognitive function [29,30,31]. The four categories of microstates reflect activities in different neural networks related to phonological, visual, salience, and attention processing, respectively [29,32]. Microstate analysis has been used to unravel the dynamic mechanisms of brain networks associated with a variety of cognitive processes and neuropsychiatric disorders, such as addiction and attention deficit hyperactivity disorder (ADHD) [33,34]. In the addictive population, researchers observed impaired functional connectivity in the salience network and attention network [35,36,37]. Therefore, analyzing resting-state EEG microstates may provide novel mechanistic insights into the neural underpinnings in non-clinical food-addicted populations.

Based on these considerations, we investigated the neurocognitive mechanisms linking attention bias and response inhibition in food addiction. We hypothesized that food-addicted individuals exhibit abnormal ERP—particularly the attention-related components—and altered resting-state attention networks. We also evaluated the extent of impairment in non-food-specific response inhibition among non-clinical food addiction individuals. Twenty overweight individuals with food addiction behaviors and twenty-three healthy control subjects were recruited. EEG data were collected during the resting state and during a food cue task to identify biomarkers of food addiction. Modified Go/No-Go tasks were used to assess response inhibition in individuals with food addiction behaviors. Our study aims to identify early neurobiological markers predictive of response inhibition impairments in food addiction.

## 2. Materials and Methods

### 2.1. Participants

We recruited 43 students from the Beijing Institute of Technology via advertisements, including 20 in the food addiction (FA) group and 23 in the healthy control (HC) group. The sample size was estimated using GPower software 3.1 to meet the significance level of p < 0.05, power of 0.8, and effect size of 0.9. The experimental procedures were approved by the Ethics Committee at the Beijing Institute of Technology (IRB/ethics board protocol number: BIT-EC-H-2023183), and all participants provided written informed consent before participating in the experiment.

The modified Yale Food Addiction Scale 2.0 (mYFAS 2.0) is a reliable tool for both clinical and non-clinical populations [38]. It has demonstrated good internal consistency, with a Cronbach’s alpha of 0.78 and a coefficient Omega of 0.92 [39]. The c-mYFAS 2.0 is a self-report questionnaire designed to assess the presence and severity of addiction-like eating behaviors. It consists of 11 symptom-related items and two diagnostic items that evaluate various aspects of food addiction, including cravings, continued consumption despite negative consequences, tolerance, withdrawal, and the inability to cut back [40,41]. Moreover, the Chinese version of the mYFAS 2.0 has been rigorously tested for reliability and validity among Chinese populations, particularly in college students, with a test-retest intraclass correlation coefficient of 0.857 [42]. The FA group was defined as individuals with a BMI of at least 25 and a positive response on at least two symptom items (without meeting a diagnostic criteria) on the c-mYFAS 2.0. Those who have a BMI within the normal weight range of 18.5 to 25 and without a current indication of food addiction as determined by the c-mYFAS 2.0 were recruited into the HC group. Any participants who had drug or alcohol abuse, anorexia, bulimia, or mental and eating disorders were excluded through verbal interviews.

### 2.2. Experimental Design and Task Paradigm

**Figure 1:**
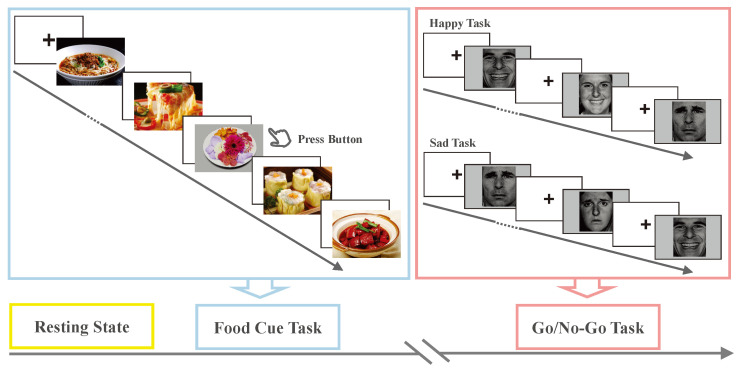
Flowchart of the experiment.

The experimental design and task paradigm are illustrated in Figure 1. Participants fasted for 4 h prior to the experiment to induce a hunger state [43], thereby accentuating attentional biases [44]. EEG data were recorded with a SynAmps RT amplifier (1000 Hz) using 64 electrodes (10–20 system), and tasks were programmed in E-Prime 3.0. The study comprised three steps. First, a two-minute resting-state EEG was collected to investigate microstates. Next, a food cue task was administered to assess attentional processing in response to food stimuli. This task involved two blocks of 90 trials (75 food images and 15 flower images from the CROCUFID database [45]), with participants required to press a key when a flower appeared [46]. Each trial presented a fixation for 1 s followed by an image for 2 s.

Finally, the Go/No-Go task evaluated response inhibition [47]. Although food-addicted individuals exhibit impaired response inhibition in food-specific contexts, their general inhibitory control appears intact [11]. However, given that previous research has shown that negative emotions (e.g., stress, sadness) can trigger binge eating [44,48], we included an emotional version of the Go/No-Go task based on the Ekman Emotional Face Database [49]. The happy/sad Go/No-Go task operates in an intermediate zone between food-specific and general response inhibition paradigms. This design mitigates floor/ceiling effects by avoiding extreme cases where there are impaired or ineffective measurements. This version of the Go/No-Go task is specifically aimed at non-clinical food addicts. In the happy version, participants responded (Go) to happy faces and withheld responses (No-Go) to sad faces; the instructions were reversed in the sad version. Each version consisted of 100 trials (7:3 Go/No-Go ratio), with each trial presenting a 1-second fixation followed by a 0.8-second stimulus. The task order was randomized.

### 2.3. EEG Microstate Analysis

For resting-state EEG data, a finite impulse response (FIR) filter was used with a range of 1–40 Hz. After averaging re-reference, the EEG data were divided into 1-second segments, with segments exceeding ±100 µV being removed, retaining 90 s of the data for each individual. Independent component analysis (ICA) was applied to detect eye movements and muscle artifacts. The Multiple Artifact Rejection Algorithm (MARA), an automated component rejection algorithm, rejected artifact components with a probability exceeding 0.5. Finally, the EEG data were downsampled to 500 Hz.

Microstate analysis was applied to the preprocessed data of resting-state EEG by the MATLAB 2021b toolbox + microstate [50]. The global field power (GFP), calculated with the standard deviation of voltage values from all electrodes on the scalp at a given time point, was used to characterize global brain activity. The formula for calculating GFP is shown as Equation (1).

(1) $$GFP_{\left(t\right)}=\sqrt{\frac{\sum_{i=1}^{M}{\left[v_{i}\left(t\right)-\bar{v}\left(t\right)\right]}^{2}}{M}}$$

where M represents the total number of channels, t is the time point, v is the potential, and $\bar{v}$ is the mean potential at all channels.

Selecting the time points of GFP peaks and ignoring the polarity, microstate segmentation was clustered using K-means. The group-level optimal number of microstates (k) was determined via a cross-validation approach. After deriving this single set of group-level microstate templates for both groups of subjects, the individual data were then fitted to these templates to compute the Global Explained Variance (GEV) for each subject. This measured how well the common templates explain the variance in each subject’s data, thereby evaluating the templates’ explanatory power and their suitability across both groups. The microstate parameters include coverage, occurrence, duration, and transition probability. Coverage indicates the proportion of time that a specific microstate dominates. Occurrence reflects the average number of appearances for a microstate per second. Duration represents the time that a microstate remains stable for the average length of time. The transitions between microstates are nonrandom, and the transition probability represents the probability of transition from one microstate to another.

### 2.4. ERP Analysis

For EEG data obtained from food cue reactivity, 75 trials including food images in each block were extracted. A passband range of 0.5–45 Hz with an FIR filter was applied. The bilateral mastoid channels (M1 and M2) were chosen as re-reference electrodes. MARA was employed to remove artifacts with a probability exceeding 0.5. Food-image epochs were merged into one dataset with 150 trials for each individual. Each trial was intercepted from pre-stimulus onset 100 ms as baseline to post-stimulus onset 800 ms. ERP waveforms were observed through the grand average across trials. ERP components involve a series of positive and negative voltage peaks within the time windows, which represent the brain’s response to food cues.

### 2.5. Statistical Analysis

Statistical analyses were conducted using IBM SPSS statistical analysis software (version 27; IBM Corp., Armonk, NY, USA). The continuous variables were compared between groups using the *t*-test if normally distributed and the Mann–Whitney test if not. The categorical variables were compared using the $X^{2}$ test.

The microstate parameters (i.e., coverage, occurrence, duration) were separately compared using a two-way analysis of variance (ANOVA) with the microstate class and group serving as two factors. When a significant effect of interaction was observed, post hoc comparisons were performed with Bonferroni correction. Cohen’s d values from the *t*-test and $\eta_{p}^{2}$ values from the F-test were measured as effect sizes [51]. Spearman analyses were performed to determine the correlations between EEG characteristics (i.e., microstate parameters, P100 amplitudes) and Go/No-Go performance (i.e., Go accuracy, No-Go accuracy, reaction time) for each group. *p*-values were corrected using the false discovery rate (FDR).

## 3. Results

### 3.1. Demographic Characteristics

As shown in Table 1, there were no significant differences in demographic data between the two groups.

### 3.2. Behavioral Test

**Figure 2:**
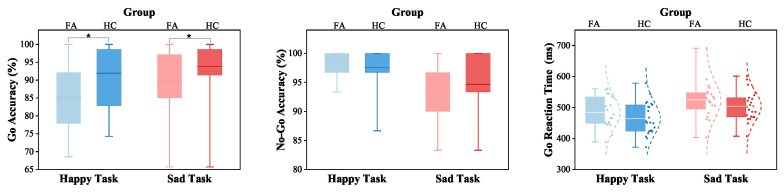
Go/No-Go task results. Box plots depict the interquartile ranges of Go accuracy, No-Go accuracy, reaction time with the maximum and minimum (cap lines), the mean (white line), the data points (dots), the normal distribution (dashed line) illustrated. * p< 0.05.

The Go accuracy, No-Go accuracy, and reaction time of Go responses in the happy and sad Go/No-Go tasks were analyzed for each participant (Figure 2). The Go or No-Go accuracy was calculated as a percentage of correct responses. The reaction time was the average response time for Go trials. For both the happy and sad tasks, the Mann–Whitney test showed reduced Go accuracy in the FA group compared to the HC group ($p_{happy}$ = 0.029, $p_{sad}$ = 0.049); however, no significant group differences in No-Go accuracy were observed ($p_{happy}$ = 0.404, $p_{sad}$ = 0.383). In the happy task, the FA group exhibited increased reaction time with a mean value of 20.08 ms, compared to the HC group. However, the group comparisons of reaction time in neither the happy task ($t_{41}$ = 1.293, p = 0.203, Cohen’s d = 0.395) nor the sad task ($t_{41}$ = 1.293, p = 0.203, Cohen’s d = 0.395) reached statistical significance. The Cohen’s d values suggest a small effect size, indicating that despite the lack of statistical significance, there may still be meaningful differences between the groups.

### 3.3. EEG Microstate Characteristics

**Figure 3:**
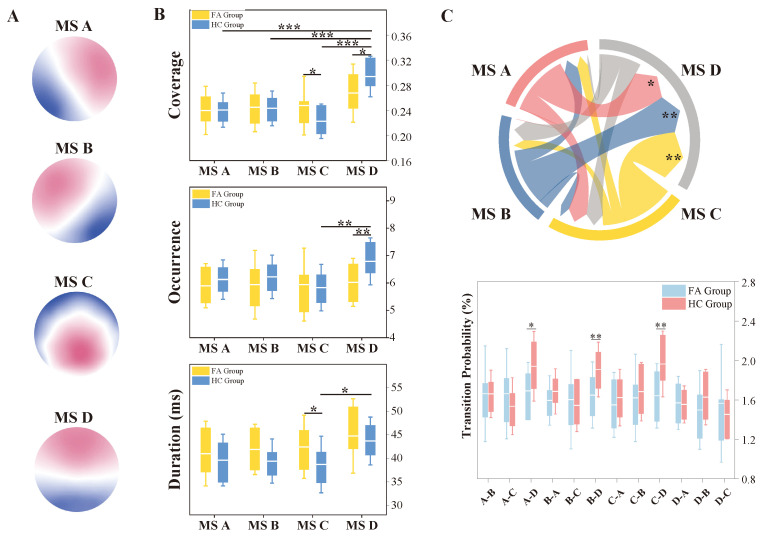
EEG microstate. (**A**) Topography maps for microstates A, B, C, and D. (**B**) Box plots depict the interquartile ranges of coverage, occurrence, and duration, with the SD (cap line) and the mean (white line). (**C**) String diagram displays the absolute difference values in transition probabilities between the two groups. Box plots illustrate the interquartile ranges of transition probabilities, with the SD (cap line) and the mean (white line). MS A, microstate A; MS B, microstate B; MS C, microstate C; MS D, microstate D. * *p* < 0.05, ** *p* < 0.01, *** *p* < 0.001.

The template maps for two groups were clustered into microstate classes A, B, C, and D, with the optimal number of clusters (k = 4) determined via cross-validation (Figure 3A). The global explained variance (GEV) represents the proportion of the total variance in EEG data that could be explained by the template maps. The GEV is 67.45 ± 4.95% for the FA group and 65.62 ± 6.14% for the HC group. Comparisons of coverage, occurrence, and duration between the two groups are visually represented in Figure 3B. As displayed in the Figure, a significant main effect in microstate class (F = 14.358, p < 0.001, $\eta_{p}^{2}$ = 0.208) and an interaction effect on coverage (F = 3.665, p = 0.014, $\eta_{p}^{2}$ = 0.063) were observed. Post hoc comparisons failed to detect significant differences among the four microstate classes within the FA group. However, the coverage of microstate D was significantly higher than that of microstates A (p < 0.001), B (p < 0.001), and C (p < 0.001) in the HC group, indicating that microstate D occurs with a higher percentage of the time. Moreover, the FA group demonstrated greater coverage of microstate C (p = 0.024) and less coverage of microstate D (p = 0.018) compared to the HC group. These findings suggest that the coverage of microstates C and D differs markedly between the two groups.

In the analysis of occurrence, the main effect of group was significant (F = 4.036, p = 0.046, $\eta_{p}^{2}$ = 0.024). However, the main effect of microstate class (F = 2.321, p = 0.077, $\eta_{p}^{2}$ = 0.041) and the interaction effect (F = 1.526, p = 0.210, $\eta_{p}^{2}$ = 0.027) did not reach a significant level. A post hoc within-group comparison in the HC group showed a significantly higher occurrence of microstate D than that of microstate C (p = 0.005), while a between-group comparison showed a significantly lower occurrence of microstate D in the FA group compared to the HC group (p = 0.009).

When analyzing the duration, a significant main effect of microstate was observed (F = 4.048, p = 0.008, $\eta_{p}^{2}$ = 0.069). There was also a significant main effect of group in microstate duration (F = 5.559, p = 0.02, $\eta_{p}^{2}$ = 0.033). However, the interaction effect did not reach statistical significance (F = 0.433, p = 0.730, $\eta_{p}^{2}$ = 0.008), suggesting that the group difference in microstate duration is not influenced by the interaction between other variables. Post hoc comparisons indicate that the duration of microstate C in the FA group was significantly longer than that in the HC group (p = 0.034). A within-group comparison in the HC group indicated a significantly shorter duration of microstate C than that of microstate D (p = 0.044), while a such difference was not observed in the FA group. These results highlight a differential duration pattern for microstates C and D between two groups.

The analysis of transition probabilities between microstates, as illustrated in Figure 3C, showed that the inter-group difference was significant for transitions to microstate D from the other three. Specifically, there was a reduction in the transition frequency of the FA group compared to the HC group from microstate A to D ($t_{41}$ = −2.505, p =0.016, Cohen’s d = 0.766), from microstate B to D ($t_{41}$ = −2.770, p = 0.008, Cohen’s d = 0.847), and from microstate C to D ($t_{41}$ = −3.182, p = 0.003, Cohen’s d = 0.973). These effect sizes highlight robust differences in the transition patterns between the groups. Particularly, the transitions to microstate D serve as a key indicator that differentiates the FA group from the HC group.

### 3.4. P100 Component

**Figure 4:**
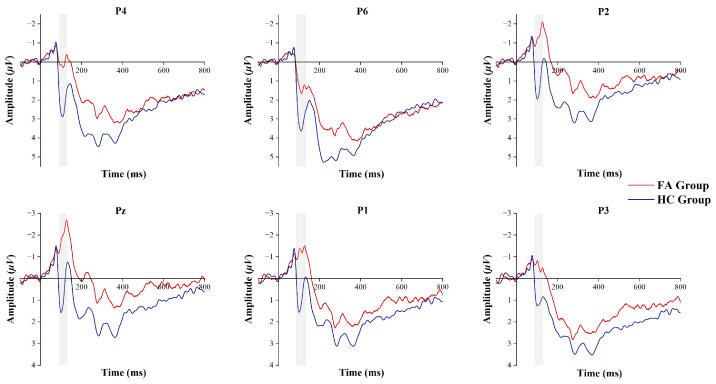
ERP evoked by food cues in the FA group and the HC group within the time window of the P100 component (shaded area).

ERP components, which were measured during brain responses to specific tasks, such as food cue stimuli, were utilized to detect neural dynamics during visual processing. Specifically, the P100 component, a key indicator of early visual processing, was calculated by averaging the voltage amplitudes within the 90–130 ms time window following the stimulus onset. As illustrated in Figure 4, the analysis revealed a significant difference in the P100 component between the two groups. The P100 amplitudes exhibited a noticeable reduction at the parietal electrodes in the FA group compared to the HC group, i.e., P6 ($t_{41}$ = −2.153, p = 0.037, Cohen’s d = 0.658), P4 ($t_{41}$ = −2.566, p = 0.021, Cohen’s d = 0.784), P2 ($t_{41}$ = −2.895, p = 0.018, Cohen’s d = 0.885), Pz ($t_{41}$ = −3.030, p = 0.018, Cohen’s d = 0.926), P1 ($t_{41}$ = −2.581, p = 0.021, Cohen’s d = 0.789), and P3 ($t_{41}$ = −2.239, p = 0.037, Cohen’s d = 0.685).

### 3.5. Correlations Between EEG Characteristics and Behavioral Performance

**Figure 5:**
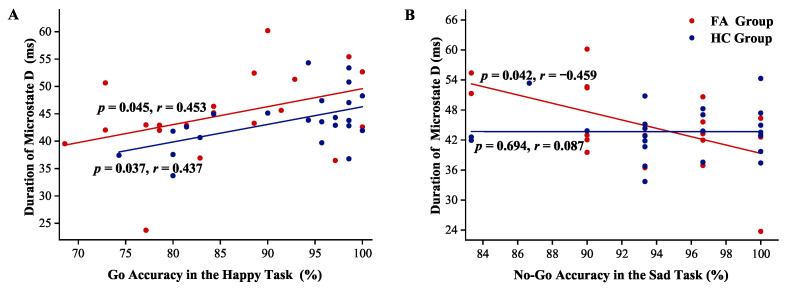
Spearman correlation between the duration of microstate D and the task performance outcome. (**A**) Correlation between the duration of microstate D and Go accuracy in the happy task. (**B**) Correlation between the duration of microstate D and No-Go accuracy in the sad task.

**Figure 6:**
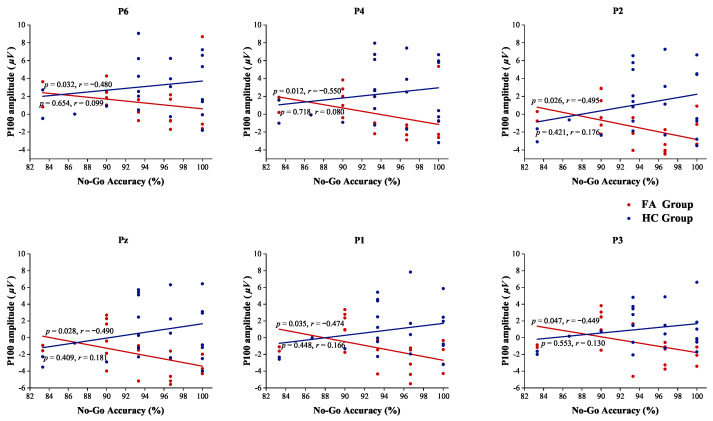
Spearman correlation between P100 amplitude and No-Go accuracy in the sad task.

During the happy Go/No-Go task, the duration of microstate D was found to be positively correlated with Go accuracy for both the groups, as shown in Figure 5A. However, a contrasting situation emerged in the sad Go/No-Go task where the duration of microstate D was negatively correlated with No-Go accuracy for the FA group (Figure 5B). In the sad Go/No-Go task, P100 amplitudes at the parietal and central channels P6, P4, P2, Pz, P1, P3 were also negatively correlated with No-Go accuracy in the FA group (Figure 6). These correlations were not observed in the HC group, highlighting a divergence in the neural and behavioral associations between the two groups. And also, P100 amplitudes did not exhibit any significant correlations with behavioral performances, such as Go accuracy, No-Go accuracy, or reaction time, during the happy Go/No-Go task in both the groups.

## 4. Discussion

This study investigated the neurophysiological and network mechanisms underlying attention and inhibitory control deficits in individuals with food addiction behaviors. Food-addicted individuals exhibited abnormal P100 components in response to food cues, reflecting early attentional processing deficits. Additionally, resting-state EEG analysis revealed disruptions in the attention network, specifically in microstate D. Importantly, with regard to non-food-specific response inhibition, we found significant correlations between neurobiological markers and response inhibition behavior in non-clinical food-addicted individuals. These findings suggest that attention abnormalities may contribute to food addiction, and that neurobiological markers may serve as early indicators of response inhibition deficits in this population.

EEG captures both spatial and temporal features, making it particularly useful for underlying mechanisms of cognitive functions [52]. EEG microstate analysis provides insights into the dynamic activity of brain networks. It has been widely applied to study the neural and psychological mechanisms of disorders such as ADHD, major depressive disorder, and addiction [53,54,55,56]. Microstates B and D can also serve as EEG characteristics for identifying gaming addiction. In individuals with gaming disorder, the duration of microstate D and the transition probability from microstate B to D increases [36]. The parameters of microstate D have been used as biomarkers for smokers [57]. The duration and coverage of microstate D were negatively correlated with the Fagerstrom Test of Nicotine Dependence. Furthermore, the occurrence of microstate C decreased in the smokers [58]. In this study, EEG microstate analysis showed an increased duration of microstate C but decreased occurrence in microstate D and the probability of transition to microstate D from other microstates in the FA group. Microstate C is associated with the salience network, while microstate D is linked to the attention network. The salience network, which includes the dorsal anterior cingulate cortex (dACC) and anterior insula, plays a crucial role in interoception and in evaluating the significance of external stimuli. In individuals with obesity, particularly those exhibiting addictive eating behaviors, there is a disrupted balance between autonomic and reward processing, with neuroimaging revealing heightened activity in the salience network [59]. Additionally, these addictive behaviors impair the attention network, which primarily involves the anterior cingulate cortex and dorsolateral prefrontal cortex [60]. These disruptions in the salience and attention networks highlight the importance of targeting these systems in developing therapeutic strategies for food addiction and obesity. This study elucidated the dynamic abnormalities in the salience and attention networks of individuals exhibiting food addiction behaviors, providing new insights into the neural mechanisms underlying food addiction.

We also observed a diminished P100 amplitude in the FA group. A prior study associated reduced P100 amplitude with impaired early attention to visual stimuli [61]. Reduced P100 amplitude in this study may suggest a disturbance of attention processing in its initial stage when the FA individuals were exposed to food cues stimuli. The reduction in the P100 amplitude is not exclusively seen in individuals with food addiction, but was also observed in the context of internet gaming disorder when processing realistic versus cartoon faces [62], and in binge drinking when individuals were exposed to alcohol-related visual stimuli [63]. Attention bias is commonly observed in individuals with addiction when they are exposed to relevant cues.

In the realm of cognitive performance, the Go/No-Go task is widely used to assess attention and response inhibition, with impairments in this task commonly observed in individuals with addiction. Previous studies have confirmed that individuals with food addiction exhibit impaired response inhibition in food-specific Go/No-Go tasks. However, general response inhibition, as measured by non-food Go/No-Go tasks, does not appear to be compromised in this population [11]. In the present study, we utilized a happy/sad Go/No-Go task, which lies in an intermediate zone between food-specific and general response inhibition paradigms. This approach allowed us to avoid floor or ceiling effects and also accounted for the link between overeating behaviors and negative emotional states in individuals with obesity. Our results showed no significant group differences in No-Go accuracy or reaction time, but a difference in Go accuracy was observed. Consistent with these findings, prior studies [12,13] have not identified a general impairment of cognitive control in individuals with food addiction behaviors, whose symptoms have not yet reached the threshold for a clinical diagnosis. Moreover, the decline in Go accuracy further supports the presence of sustained attention deficits in non-clinical food addicts. These results provide additional validation for the appropriateness of our experimental design.

Our study identified a significant association between prolonged microstate D duration and reduced No-Go task accuracy under sad-task conditions in the food addiction (FA) group, a phenomenon absent in healthy controls (HC). This observation aligns with findings in chronic smokers, where extended microstate D durations correlated with cumulative smoking duration, implicating sustained attentional network activation as a potential contributor to impaired response inhibition [57]. These results suggest that abnormal attentional network dynamics in individuals with FA may predispose them to diminished inhibitory control. Notably, while the FA group exhibited attenuated P100 amplitudes to food cues compared to HC participants, we observed a paradoxical inverse relationship: higher P100 amplitudes within the FA group were associated with poorer No-Go task performance. This mirrors observations in nicotine-dependent populations, where amplified P100 responses to smoking-related stimuli during craving states reflect heightened stimulus salience [64]. This result may suggest that individuals with addiction, who exhibit an exaggerated response to food cues, show a tendency for reduced response inhibition. Collectively, these findings highlight dysregulated attentional allocation as a vulnerability factor for inhibitory deficits.

Despite meaningful results obtained, several issues need to be addressed in future studies. The inclusion of clinical populations with food addiction or eating disorders will help to verify whether the mechanistic associations between response inhibition and attention were evident in typical pathological populations of food addiction. Further, a combination of neuroimaging and EEG data will be more conducive to the analysis of the spatiotemporal dynamic mechanisms of brain networks associated with response inhibition and attention in food addiction. Finally, the identification of neurophysiological indicators and brain networks in patients with food addiction associated with attention and response inhibition in this study provide a foundation for future interventions. Targeted interventions via non-invasive neuromodulation may provide prospective validation of the mechanistic results observed in this study.

## 5. Conclusions

In conclusion, our study highlights key neural abnormalities in food addiction, including abnormal P100 components related to early attentional processing in response to food cues and disruptions in the resting-state attention network. Additionally, we found that non-food-specific response inhibition, as measured by modified Go/No-Go tasks, remains intact in food-addicted individuals. Furthermore, the correlation between neurobiological markers and response inhibition behavior in non-clinical food-addicted individuals provides valuable insights into early biomarkers of food addiction. These findings offer important implications for developing targeted interventions aimed at enhancing attention control and self-regulation in food addiction, potentially reducing associated compulsive behaviors.

## Figures and Tables

**Table 1:** Demographic characteristics of participants.

	FA Group (n = 20)	HC Group (n = 23)	*p* Value
Gender (Male/Female)	14/6	11/12	0.142
Age (years)	24.44 ± 6.26	23.18 ± 2.88	0.390
Education (years)	17.52 ± 2.75	16.47 ± 1.33	0.114
c-mYFAS 2.0	5.05 ± 2.63	0.35 ± 0.49	<0.001
BMI	28.96 ± 4.37	21.29 ± 2.00	<0.001

Note: Mean ± SD are reported. Abbreviations: FA, food addiction; HC, healthy control; c-mYFAS 2.0, Chinese version of modified Yale Food Addiction Scale 2.0; BMI, body mass index.

## Data Availability

Dataset available on request from the authors.

## References

[1] Schulte E., Gearhardt A. (2021). Attributes of the food addiction phenotype within overweight and obesity. Eat. Weight. Disord.-Stud. Anorex. Bulim. Obes..

[2] De Ridder D., Manning P., Leong S.L., Ross S., Sutherland W., Horwath C., Vanneste S. (2016). The brain, obesity and addiction: An EEG neuroimaging study. Sci. Rep..

[3] Benzerouk F., Gierski F., Ducluzeau P.H., Bourbao-Tournois C., Gaubil-Kaladjian I., Bertin É., Kaladjian A., Ballon N., Brunault P. (2018). Food addiction, in obese patients seeking bariatric surgery, is associated with higher prevalence of current mood and anxiety disorders and past mood disorders. Psychiatry Res..

[4] Pursey K.M., Stanwell P., Gearhardt A.N., Collins C.E., Burrows T.L. (2014). The prevalence of food addiction as assessed by the Yale Food Addiction Scale: A systematic review. Nutrients.

[5] Adams R.C., Sedgmond J., Maizey L., Chambers C.D., Lawrence N.S. (2019). Food addiction: Implications for the diagnosis and treatment of overeating. Nutrients.

[6] Gearhardt A. (2016). Current Considerations Regarding Food Addiction. J. Behav. Addict..

[7] Lawrence N.S., Porter L., Staiger P.K. (2022). The ‘Go’s and the ‘No-Go’s of response-inhibition training to food: Lessons learned from trials. Curr. Opin. Behav. Sci..

[8] Camp B., Lawrence N.S. (2019). Giving pork the chop: Response inhibition training to reduce meat intake. Appetite.

[9] McGreen J., Kemps E., Tiggemann M. (2024). The effectiveness of Go/No-Go and Stop-Signal training in reducing food consumption and choice: A systematic review and meta-analysis. Appetite.

[10] Liu Y., Roefs A., Nederkoorn C. (2022). Fluctuations in attentional bias for food and the role of executive control. Appetite.

[11] Price M., Lee M., Higgs S. (2016). Food-specific response inhibition, dietary restraint and snack intake in lean and overweight/obese adults: A moderated-mediation model. Int. J. Obes..

[12] Hardee J.E., Phaneuf C., Cope L., Zucker R., Gearhardt A., Heitzeg M. (2020). Neural correlates of inhibitory control in youth with symptoms of food addiction. Appetite.

[13] Meule A., Lutz A., Vögele C., Kübler A. (2012). Women with elevated food addiction symptoms show accelerated reactions, but no impaired inhibitory control, in response to pictures of high-calorie food-cues. Eat. Behav..

[14] Stice E., Yokum S., Nelson T.D., Berkman E., Veling H., Lawrence N. (2022). Efficacy of a combined food-response inhibition and attention training for weight loss. Curr. Opin. Behav. Sci..

[15] Seo C.L., Lee J.H. (2021). Attentional bias to high-calorie food in binge eaters with high shape/weight concern. Front. Psychiatry.

[16] Davis C. (2010). Attention-deficit/hyperactivity disorder: Associations with overeating and obesity. Curr. Psychiatry Rep..

[17] Schulte E.M., Yokum S., Jahn A., Gearhardt A.N. (2019). Food cue reactivity in food addiction: A functional magnetic resonance imaging study. Physiol. Behav..

[18] Khajehpour H., Mohagheghian F., Bakht S., Samadzadehaghdam N., Eqlimi E., Makkiabadi B. (2019). Event-related potential correlates of biased cognitive processing and control in substance abusers: A review. Front. Biomed. Technol..

[19] Clark V.P., Fan S., Hillyard S.A. (1994). Identification of early visual evoked potential generators by retinotopic and topographic analyses. Hum. Brain Mapp..

[20] Aviram-Friedman R., Kafri L., Baz G., Alyagon U., Zangen A. (2020). Prisoners of addictive cues: Biobehavioral markers of overweight and obese adults with food addiction. Nutrients.

[21] Hofmann J., Ardelt-Gattinger E., Paulmichl K., Weghuber D., Blechert J. (2015). Dietary restraint and impulsivity modulate neural responses to food in adolescents with obesity and healthy adolescents. Obesity.

[22] Fehr T., Wiedenmann P., Herrmann M. (2006). Nicotine Stroop and addiction memory—An ERP study. Int. J. Psychophysiol..

[23] Donohue S.E., Woldorff M.G., Hopf J.M., Harris J.A., Heinze H.J., Schoenfeld M.A. (2016). An electrophysiological dissociation of craving and stimulus-dependent attentional capture in smokers. Cogn. Affect. Behav. Neurosci..

[24] Petit G., Kornreich C., Maurage P., Noël X., Letesson C., Verbanck P., Campanella S. (2012). Early attentional modulation by alcohol-related cues in young binge drinkers: An event-related potentials study. Clin. Neurophysiol..

[25] Blume M., Schmidt R., Hilbert A. (2019). Abnormalities in the EEG power spectrum in bulimia nervosa, binge-eating disorder, and obesity: A systematic review. Eur. Eat. Disord. Rev..

[26] Han X.D., Zhang H.W., Xu T., Liu L., Cai H.T., Liu Z.Q., Li Q., Zheng H., Xu T., Yuan T.F. (2022). How impulsiveness influences obesity: The mediating effect of resting-state brain activity in the dlPFC. Front. Psychiatry.

[27] Michaud A., Vainik U., Garcia-Garcia I., Dagher A. (2017). Overlapping neural endophenotypes in addiction and obesity. Front. Endocrinol..

[28] Li Y., Gao J., Yang Y., Zhuang Y., Kang Q., Li X., Tian M., Lv H., He J. (2024). Temporal and spatial variability of dynamic microstate brain network in disorders of consciousness. CNS Neurosci. Ther..

[29] Britz J., Van De Ville D., Michel C.M. (2010). BOLD correlates of EEG topography reveal rapid resting-state network dynamics. Neuroimage.

[30] Gao F., Jia H., Wu X., Yu D., Feng Y. (2017). Altered resting-state EEG microstate parameters and enhanced spatial complexity in male adolescent patients with mild spastic diplegia. Brain Topogr..

[31] Chen J., Ke Y., Ni G., Liu S., Ming D. (2023). Evidence for modulation of EEG microstates by mental workload levels and task types. Hum. Brain Mapp..

[32] Qiu S., Lyu X., Zheng Q., He H., Jin R., Peng W. (2023). Temporal dynamics of electroencephalographic microstates during sustained pain. Cerebral Cortex.

[33] Ding X.B., Li X.Y., Xu M., He Z.J., Jiang H. (2023). The effect of repetitive transcranial magnetic stimulation on electroencephalography microstates of patients with heroin-addiction. Psychiatry Res. Neuroimaging.

[34] Férat V., Arns M., Deiber M.P., Hasler R., Perroud N., Michel C.M., Ros T. (2022). Electroencephalographic microstates as novel functional biomarkers for adult attention-deficit/hyperactivity disorder. Biol. Psychiatry Cogn. Neurosci. Neuroimaging.

[35] Cushnie A.K., Tang W., Heilbronner S.R. (2023). Connecting Circuits with Networks in Addiction Neuroscience: A Salience Network Perspective. Int. J. Mol. Sci..

[36] Wang L., Ding X., Zhang W., Yang S. (2021). Differences in EEG microstate induced by gaming: A comparison between the gaming disorder individual, recreational game users and healthy controls. IEEE Access.

[37] Li H., Yue J., Wang Y., Zou F., Zhang M., Wu X. (2021). Negative effects of mobile phone addiction tendency on spontaneous brain microstates: Evidence from resting-state EEG. Front. Hum. Neurosci..

[38] Brunault P., Berthoz S., Gearhardt A.N., Gierski F., Kaladjian A., Bertin E., Tchernof A., Biertho L., de Luca A., Hankard R. (2020). The modified Yale Food Addiction Scale 2.0: Validation among non-clinical and clinical French-speaking samples and comparison with the full Yale Food Addiction Scale 2.0. Front. Psychiatry.

[39] Escrivá-Martínez T., Galiana L., Herrero R., Rodríguez-Arias M., Fernández-Aranda F., Gearhardt A.N., Baños R.M. (2023). Food addiction and its relationship with other eating behaviours among Spanish university students. J. Eat. Disord..

[40] Li S., Schulte E.M., Cui G., Li Z., Cheng Z., Xu H. (2022). Psychometric properties of the Chinese version of the modified Yale Food Addiction Scale version 2.0 (C-mYFAS 2.0): Prevalence of food addiction and relationship with resilience and social support. Eat. Weight. Disord.-Stud. Anorex. Bulim. Obes..

[41] Schulte E.M., Gearhardt A.N. (2017). Development of the modified Yale food addiction scale version 2.0. Eur. Eat. Disord. Rev..

[42] Zhang H., Tong T., Gao Y., Liang C., Yu H., Li S., Yan X., Wang L. (2021). Translation of the Chinese version of the modified Yale Food Addiction Scale 2.0 and its validation among college students. J. Eat. Disord..

[43] Tammela L.I., Pääkkönen A., Karhunen L.J., Karhu J., Uusitupa M.I., Kuikka J.T. (2010). Brain electrical activity during food presentation in obese binge-eating women. Clin. Physiol. Funct. Imaging.

[44] Lobera I.J. (2012). Neurophysiological Basis of Food Craving. State of the Art of Therapeutic Endocrinology.

[45] Toet A., Kaneko D., De Kruijf I., Ushiama S., Van Schaik M.G., Brouwer A.M., Kallen V., Van Erp J.B.F. (2019). CROCUFID: A cross-cultural food image database for research on food elicited affective responses. Front. Psychol..

[46] Bu J., Young K.D., Hong W., Ma R., Song H., Wang Y., Zhang W., Hampson M., Hendler T., Zhang X. (2019). Effect of deactivation of activity patterns related to smoking cue reactivity on nicotine addiction. Brain.

[47] Cragg L., Nation K. (2008). Go or no-go? Developmental improvements in the efficiency of response inhibition in mid-childhood. Dev. Sci..

[48] Whiteside U., Chen E., Neighbors C., Hunter D., Lo T., Larimer M. (2007). Difficulties regulating emotions: Do binge eaters have fewer strategies to modulate and tolerate negative affect?. Eat. Behav..

[49] Boucher J.D., Ekman P. (1975). Facial areas and emotional information. J. Commun..

[50] Tait L., Zhang J. (2022). +microstate: A MATLAB toolbox for brain microstate analysis in sensor and cortical EEG/MEG. Neuroimage.

[51] Cohen J. (2013). Statistical Power Analysis for the Behavioral Sciences.

[52] Ye W., Wang J., Chen L., Dai L., Sun Z., Liang Z. (2024). Adaptive Spatial-Temporal Aware Graph Learning for EEG-Based Emotion Recognition. Cyborg Bionic Syst..

[53] Lehmann D., Faber P.L., Galderisi S., Herrmann W.M., Kinoshita T., Koukkou M., Mucci A., Pascual-Marqui R.D., Saito N., Wackermann J. (2005). EEG microstate duration and syntax in acute, medication-naive, first-episode schizophrenia: A multi-center study. Psychiatry Res. Neuroimaging.

[54] Baradits M., Bitter I., Czobor P. (2020). Multivariate patterns of EEG microstate parameters and their role in the discrimination of patients with schizophrenia from healthy controls. Psychiatry Res..

[55] Luo N., Luo X., Zheng S., Yao D., Zhao M., Cui Y., Zhu Y., Calhoun V.D., Sun L., Sui J. (2023). Aberrant brain dynamics and spectral power in children with ADHD and its subtypes. Eur. Child Adolesc. Psychiatry.

[56] Lei L., Liu Z., Zhang Y., Guo M., Liu P., Hu X., Yang C., Zhang A., Sun N., Wang Y. (2022). EEG microstates as markers of major depressive disorder and predictors of response to SSRIs therapy. Prog. Neuro-Psychopharmacol. Biol. Psychiatry.

[57] Gan H., Bu J., Zeng G.Q., Gou H., Liu M., Cui G., Zhang X. (2023). Correlation between abnormal brain network activity and electroencephalogram microstates on exposure to smoking-related cues. BJPsych Open.

[58] Li X., Dong F., Zhang Y., Wang J., Wang Z., Sun Y., Zhang M., Xue T., Ren Y., Lv X. (2022). Altered resting-state electroencephalography microstate characteristics in young male smokers. Front. Psychiatry.

[59] García-García I., Jurado M.Á., Garolera M., Segura B., Sala-Llonch R., Marqués-Iturria I., Pueyo R., Sender-Palacios M.J., Vernet-Vernet M., Narberhaus A. (2013). Alterations of the salience network in obesity: A resting-state fMRI study. Hum. Brain Mapp..

[60] Visintin E., Panfilis C.D., Antonucci C., Capecci C., Marchesi C., Sambataro F. (2015). Parsing the intrinsic networks underlying attention: A resting state study. Behav. Brain Res..

[61] Shen I.H., Tsai S.Y., Duann J.R. (2011). Inhibition control and error processing in children with attention deficit/hyperactivity disorder: An event-related potentials study. Int. J. Psychophysiol..

[62] He J., Zheng Y., Fan L., Pan T., Nie Y. (2019). Automatic processing advantage of cartoon face in internet gaming disorder: Evidence from P100, N170, P200, and MMN. Front. Psychiatry.

[63] Petit G., Kornreich C., Dan B., Verbanck P., Campanella S. (2014). Electrophysiological correlates of alcohol-and non-alcohol-related stimuli processing in binge drinkers: A follow-up study. J. Psychopharmacol..

[64] Mashhoon Y., Betts J., Farmer S.L., Lukas S.E. (2018). Early onset tobacco cigarette smokers exhibit deficits in response inhibition and sustained attention. Drug Alcohol Depend..

